# Hybrid positron emission tomography–magnetic resonance of the heart: current state of the art and future applications

**DOI:** 10.1093/ehjci/jey090

**Published:** 2018-07-12

**Authors:** Muhummad Sohaib Nazir, Tevfik F Ismail, Eliana Reyes, Amedeo Chiribiri, Philipp A Kaufmann, Sven Plein

**Affiliations:** 1 School of Biomedical Engineering and Imaging Sciences, King's College London, St Thomas' Hospital, Westminster Bridge Road, London, UK; 2 Department of Nuclear Medicine, Cardiac Imaging, University Hospital Zurich, Ramistrasse 100, Zurich, Switzerland; 3 Leeds Institute of Cardiovascular and Metabolic Medicine, LIGHT Laboratories, Clarendon Way, University of Leeds, Leeds, UK

**Keywords:** cardiovascular magnetic resonance, cardiovascular positron emission tomography, hybrid imaging, MR-PET, PET-MR, PET-MRI

## Abstract

Hybrid positron emission tomography–magnetic resonance (PET-MR) imaging is a novel imaging modality with emerging applications for cardiovascular disease. PET-MR aims to combine the high-spatial resolution morphological and functional assessment afforded by magnetic resonance imaging (MRI) with the ability of positron emission tomography (PET) for quantification of metabolism, perfusion, and inflammation. The fusion of these two modalities into a single imaging platform not only represents an opportunity to acquire complementary information from a single scan, but also allows motion correction for PET with reduction in ionising radiation. This article presents a brief overview of PET-MR technology followed by a review of the published literature on the clinical cardio-vascular applications of PET and MRI performed separately and with hybrid PET-MR.

## Introduction

Positron emission tomography (PET) and magnetic resonance imaging (MRI) are well established modalities for the investigation of cardiovascular disease. MRI provides high-resolution information on anatomy, morphology, function, and tissue characteristics. Parametric mapping with MRI allows visualization of quantitative changes in the myocardium based on changes in T1, T2, T2* and allows detection of myocardial fibrosis, infiltration, and inflammation and iron overload.^1^ PET allows assessment of physiological processes by labelling biological compounds with positron emitting radionuclides^2^ and is the reference standard for non-invasive assessment of myocardial perfusion and absolute myocardial blood flow (MBF), myocardial metabolism, and inflammation. Integrated PET and MRI may confer synergistic value from image co-registration, motion correction, and reduction in ionising radiation for clinical applications.

This article presents a brief overview of positron emission tomography–magnetic resonance (PET-MR) technology followed by a review of the published literature on the clinical cardio-vascular applications of PET and MRI performed separately and with hybrid PET-MR.

## Technical considerations

Simultaneous PET-MR was first demonstrated in preclinical phantom models in 1997.^2^ Presently, two configurations of PET-MR systems exist for clinical studies. The Philips Ingenuity PET-MR (Philips Medical Systems, the Netherlands) allows sequential imaging with a turntable aligned to the PET and MRI gantry. The Siemens Biograph mMR PET-MR (Siemens Healthcare, Germany) and GE Signa PET-MR (GE Healthcare, Waukesha) are fully integrated systems in the same gantry for true simultaneous PET and MRI acquisition.

The integration of PET and MRI systems in a single hardware platform presented several technical challenges.^2^ A major obstacle was the production of MRI compatible PET detectors that could operate safely and efficiently within an MRI scanner. In addition, rapidly alternating gradient fields of an MRI scanner could induce eddy currents in PET circuitry and lead to signal loss, heating, and vibration.^2^ MRI radiofrequency pulses cause electronic interference with standard PET detectors. On the other hand, standard ferromagnetic PET detectors may cause inhomogeneities in the magnetic field and lead to susceptibility artefacts and degrade MRI quality. Innovative solutions overcame these challenges, particularly the development of PET detectors that operate within a strong magnetic field.^2^

### Attenuation correction

Quantitative PET data requires an accurate method for photon attenuation, which occurs when photons that undergo annihilation are absorbed by the body. As such, PET pixel values need to be scaled to radioactivity concentrations units.^3^ In PET-CT, attenuation correction (AC) of PET data is derived from computed tomography (CT) data, which are rescaled from Hounsfield units to 511-keV linear attenuation coefficients to generate robust μ-maps.^4^ In PET-MR, AC is challenging as MRI signal intensity reflects proton density, which has no direct relationship with tissue density or photon absorption as do CT data.^5^ In PET-MR, attenuation maps are commonly derived from Dixon MRI sequences,^6^ which are then used to segment discrete anatomical regions into air, lung, fat, or soft tissue with known linear attenuation coefficients,^7^ and voxels that belong to these tissue classes are assigned a corresponding linear attenuation coefficient.^8^

Published comparisons between PET-CT and PET-MR quantitative data have demonstrated comparable standardized uptake values (SUV) of cardiac ^18^F-fluorodeoxyglucose (^18^F-FDG).^9–12^ Respiratory misalignment commonly occurs with MRI-based AC maps, and metallic artefact produces voids, but these can be corrected with manual adjustment and importantly were found not to impact quantitative PET data.^13^

However, there are several challenges to AC with PET-MR, in particular, cortical bone (the tissue that contributes the greatest to attenuation) and air have low MRI signal, yet both are at the extremes of photon attenuation. Standard Dixon based MRI-based AC maps do not consider bone and solutions have been proposed with a Dixon based model-based bone segmentation algorithm^14^ and ultrashort echo time (UTE) sequences.^8^^,^^15^

### Motion correction

Hybrid PET-MR has potential to provide high-resolution MRI data with excellent soft-tissue contrast to track respiratory movement to motion-correct PET data.^16^ Multiple radiation-free MRI acquisitions can be acquired simultaneously to enhance image quality and quantitative PET data. Several studies have demonstrated significant respiratory motion correction and improvement in accuracy of quantitative SUV with MRI respiratory motion data.^17–19^ Furthermore, methods are developing for highly efficient MRI motion correction models to simultaneously correct coronary MRI and PET data with potential to enhance PET-MR imaging.^20^

## Clinical applications of PET-MR

There are many potential clinical applications for PET-MR in cardiovascular imaging, but to date, the development has focussed on a small number of applications, which are discussed below.

## Myocardial perfusion imaging

MRI and PET perfusion imaging are both in routine clinical use for ischaemia testing with similar levels of evidence in European Society of Cardiology guidelines for diagnosis of stable angina.^21^

### MRI

Myocardial perfusion MRI tracks the first myocardial passage of a gadolinium-based contrast agent (GBCA) following intravenous injection. GBCAs shorten T1-relaxation time and increase signal intensity on T1-weighted MRI pulse sequences, which are used to acquire a dynamic series of images. Typically combined with vasodilator stress, myocardial perfusion MRI for ischaemia assessment is recommended in patients with intermediate risk of coronary artery disease (CAD).^21^

In meta-analyses, myocardial perfusion MRI has a sensitivity of 87% and 89% and specificity of 91% and 87% at the vessel-level and patient-level respectively when compared with invasive fractional flow reserve as the reference standard.^22^ Absolute MBF quantification with MRI is feasible and provides independent incremental prognostic value compared with visual analysis.^23^ Techniques for fully automated, free-breathing, pixel-wise MBF quantification with MRI showed good agreement against PET, and promise to accelerate adoption into clinical practice.^24^^,^^25^

### PET

Perfusion PET tracers (*Table **1*) are generated by cyclotron (^15^O-Water, ^13^N-Ammonia, ^18^F-Flurpiridaz) or generator (Rubidium-82) and allow visual or fully quantitative assessment of myocardial perfusion at rest and stress. A meta-analysis demonstrated sensitivity 92% and specificity 85% for PET to detect angiographically defined CAD.^29^ A normal PET perfusion scan is associated with a <1% annual cardiac event rate, while an abnormal scan indicates adverse prognosis.^30^^,^^31^

**Table 1:** Physical properties of perfusion PET tracers

Radiotracer	Half-life	Availability	Mechanism	Comments
^15^O-Water	122 s	On-site cyclotron	Metabolically inert, diffuses freely across capillary membrane	Ideal tracer for MBF quantification, near perfect linear relationship between flow and tracer uptake.^26^^,^^27^ 100% myocardial extraction fraction. Intermediate image quality due to long positron range.
^13^N-Ammonia	9.96 min	On-site cyclotron	Diffusion and metabolic trapping	>80% myocardial extraction fraction. High image quality due to short positron range and myocardial retention. Validated against ^15^O-Water.^27^^,^^28^
Rubidium-82	76 s	Generator	Myocardial uptake via Na/K-ATPase	Short half-life allows rapid protocols. MBF underestimation at high-flow rates due to roll-off phenomenon. 65% myocardial extraction fraction. Moderate image quality due to long positron range.
^18^F-Flurpiridaz	110 min	Regional cyclotron	Rapid uptake by myocyte mitochondrial complex	Near linear kinetics of tracer uptake and MBF. Good image quality due to short positron range. High myocardial extraction fraction (94%). Current evaluation in Phase III trials. Does not require on-site cyclotron and therefore allows greater access.

**Table 2:** Radiotracers with potential clinical utility with PET-MR

Radiotracer	Mechanism	Potential use	Future applications
^18^F-FDG	Glucose analogue, undergoes intracellular phosphorylation and trapped without further metabolism	Viability Inflammation Sarcoidosis	Integrated ^18^F-FDG with dobutamine stress MRI, LGE, and mapping may accurately predict functional recovery. Potential for improved diagnostic accuracy and risk stratification in cardiac sarcoidosis and myocarditis with combined mapping, LGE and ^18^F-FDG.
^18^F-Flouride	Microcalcification	Coronary plaque imaging Amyloid	Fused coronary anatomy with high-risk atherosclerotic inflammatory activity with ^18^F-Fluoride may predict plaque rupture to guide preventative therapy. May discriminate ATTR amyloid from AL amyloid.
^68^Ga-DOTATATE	Binds to activated inflammatory macrophages	Coronary plaque imaging	Superior coronary imaging and better discriminator between culprit and non-culprit lesions compared with ^18^F-FDG. Combined anatomical and metabolic activity could be used to identify high-risk lesions prior to rupture.
^18^F-Florbetaben	Binds to β amyloid	Amyloid	Integrated T1-mapping and LGE with MRI with novel PET tracers may accurately diagnose cardiac amyloid.
^11^C-Acetate	Oxidative metabolism	Viability assessment Metabolic efficiency assessment	Combined ^11^C-Acetate and low-dose-dobutamine may accurately predict functional recovery following coronary revascularization or response to cardiac resynchronization therapy.

PET is the *in vivo* reference standard for MBF quantification.^32^ PET MBF is superior to visual analysis for CAD detection and improves diagnostic accuracy in multi-vessel CAD and microvascular disease.^33–35^ A reduced myocardial perfusion reserve (MPR), ratio of stress: rest MBF, is an independent marker for adverse cardiovascular outcome.^36^^,^^37^

### PET-MR

There is only one published study on the use of hybrid PET-MR for myocardial perfusion imaging. Twenty-nine patients underwent simultaneous stress and rest PET-MR perfusion with ^13^N-Ammonia and first-pass GBCA MRI.^38^ A good correlation and agreement was reported with MRI MBF compared with PET MBF, with improved correlation after haematocrit correction to convert MRI plasma flow values to blood flow. However, in keeping with previous studies comparing MRI and PET MBF, MRI tended to overestimate MBF. Potential reasons for this overestimation include the differences in quantification algorithms between the methods and the fact that the gold standard PET tracer ^15^O-Water was not used.

#### Future outlook

Although hybrid PET-MR perfusion imaging is an unlikely future clinical application as both modalities measure MBF, PET-MR is an ideal platform to cross-validate the two methods under identical haemodynamic conditions and remove physiological variation in measurement. Emerging MRI methods for MBF quantification may be compared against the gold standard PET tracer ^15^O-Water with potential to provide an alternative future method for MBF quantification that is free of ionizing radiation.

## Viability

Viability assessment is most commonly used to predict functional recovery following revascularization. Both MRI and PET are in clinical use, but take conceptually different approaches to viability/scar assessment.

### MRI

In clinical practice, MRI assessment of myocardial viability is most commonly based on late gadolinium enhancement (LGE). The distribution volume of the clinically used extracellular GBCAs is increased in infarcted tissue due to its larger extracellular volume (ECV) and slower clearance of GBCA. LGE imaging exploits these differences in contrast concentration and generates images of myocardial scar with high-tissue contrast and high-spatial resolution. On LGE, segments with <25% transmural extent of infarction have a high likelihood of functional recovery whereas segments with >75% transmural infarction are unlikely to recover after revascularization.^39^ An alternative method for viability assessment with MRI is low-dose dobutamine cine imaging, which has comparable accuracy to stress echocardiography to detect functional reserve as a marker of viability. In a meta-analysis, 50% subendocardial LGE had sensitivity (95%) and sensitivity (51%) for prediction of functional recovery following revascularization.^40^ Low-dose dobutamine had a lower sensitivity (81%) but greater specificity (91%) to predict functional recovery.^40^

### PET

Viability assessment by PET is based on metabolic assessment. ^18^F-FDG is a radiolabelled glucose analogue that becomes trapped within cardiac myocytes following intracellular uptake and provides strong signal for PET imaging.^41^^,^^42^ Optimal assessment of viability requires integration of perfusion and ^18^F-FDG uptake.^42^ Dysfunctional segments are ‘chronically stunned’ (preserved perfusion and ^18^F-FDG uptake), ‘hibernating’ (impaired perfusion, preserved ^18^F-FDG uptake), or ‘scarred’ (impaired perfusion and ^18^F-FDG uptake).^42^ A pooled analysis demonstrated sensitivity of 92% and specificity of 63% for ^18^F-FDG to predict functional recovery following revascularization.^43^

### PET-MR

As shown, MRI and PET use different biological approaches to assess myocardial viability. To date, most studies have focussed on cross-validation of techniques rather than exploitation of synergy.

**Figure 1:**
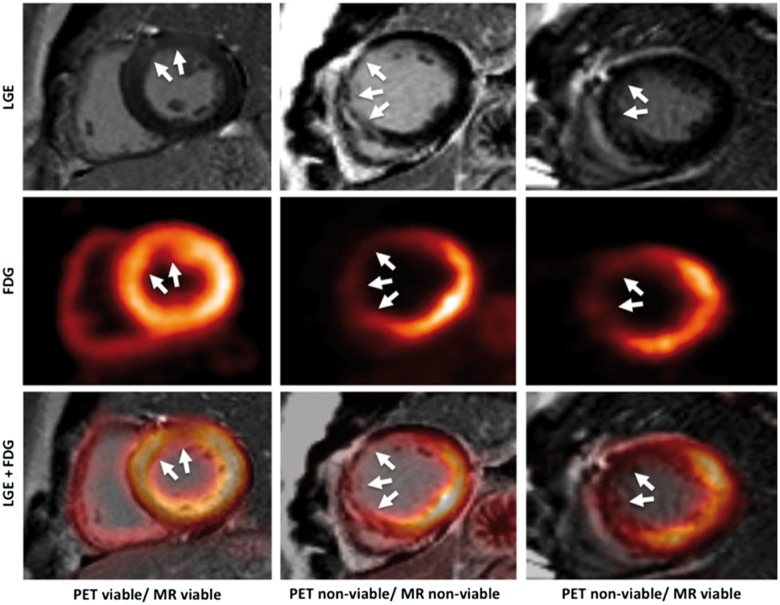
Different combinations of ^18^F-FDG uptake and LGE transmurality with PET-MR. Left column: ‘PET viable/MRI viable’; middle column: ‘PET non-viable/MRI non-viable’; right column: ‘PET non-viable/MRI viable’. White arrows indicate infarcted myocardium. Reproduced with permission from ref. 44.

Published studies have reported good agreement with LGE transmurality and ^18^F-FDG uptake with PET-MR in viability assessment.^9^^,^^44^^,^^45^ In a study of patients prior to consideration for coronary revascularization, PET-MR reclassified 19% of segments reported as ‘not assessable’ due to intermediate LGE (25–75% transmurality) after integration of ^18^F-FDG.^46^ This led to a recommendation for revascularization of only one additional coronary vessel compared with MRI alone in 12 patients.^46^ MRI performed at 6 months follow-up found that initial ^18^F-FDG was a better predictor for functional recovery than baseline LGE (*Figure **1*).^44^

#### Future outlook

Hybrid PET-MR offers potential for combined morphological, functional, and metabolic assessment of myocardial viability. Hybrid PET-MR may provide incremental diagnostic value particularly in cases where MRI or PET alone provide intermediate probabilities of functional recovery such as in cases with LGE of 25–75%. Furthermore, in complex cases such as multivessel CAD and ischaemic cardiomyopathy, multimodal imaging may provide complementary information to more accurately predict functional recovery. This could be achieved in a single PET-MR scan by combining data on contractile function with dobutamine stress cardiovascular magnetic resonance and co-registration of ^18^F-FDG PET activity in addition to wall thickness and LGE. Potential clinical utility will only be realized in larger studies that define clear incremental benefit of patient outcomes.

## Coronary imaging

Non-invasive coronary imaging is the domain of cardiac CT but both MRI and PET provide relevant information on coronary arterial pathology, in particular atherosclerotic plaque, and their combination holds promise for risk stratification.

### MRI

Magnetic resonance angiography can visualize the proximal course of coronary arteries in almost all cases^47^ and is recommended in guidelines for assessment of coronary artery anomalies and aortocoronary bypass grafts but not detection of coronary stenosis.^48^ Coronary vessel wall imaging with black-blood MRI demonstrates increased wall thickness in CAD patients.^49^^,^^50^ Non-contrast T1-weighted images visualized intracoronary thrombus with sensitivity 91% in patients following acute myocardial infarction.^51^ Furthermore, high-intensity plaques identified by non-contrast T1-weighted images were an independent factor for predicting coronary events.^52^

### PET

Arterial ^18^F-FDG and increased carotid ^18^F-FDG uptake are associated with increased risk of adverse cardiovascular events.^53^^,^^54^ However, ^18^F-FDG coronary imaging is challenging as left ventricular myocardial glucose uptake often obscures activity within coronary vessels. There is growing interest into ^18^F-Fluoride, which has a predilection for vascular microcalcification, a feature of high-risk atheroma.^55^ Coronary ^18^F-Fluoride uptake was demonstrated in patients with high Framingham risk score and was localized in culprit lesions in 93% of patients following acute myocardial infarction.^56^^,^^57^^68^Ga-DOTATATE, a specific macrophage tracer, was detected in culprit coronary lesions in patients with recent myocardial infarction or high-risk stable lesions and was a better discriminator of high-risk atherosclerotic lesions than ^18^F-FDG.^58^

### PET-MR

The potential of hybrid PET-MR for coronary imaging has been demonstrated in a number of feasibility studies.

Coronary PET-MR was performed with ^18^F-Fluoride or ^18^F-FDG in 23 patients with CAD or risk factors for CAD.^59^ Several ‘hotspots’ of ^18^F-Fluoride were observed in the coronary arteries of patients with established CAD and a novel AC method eliminated significant artefacts compared with standard AC methods.^59^

**Figure 2:**
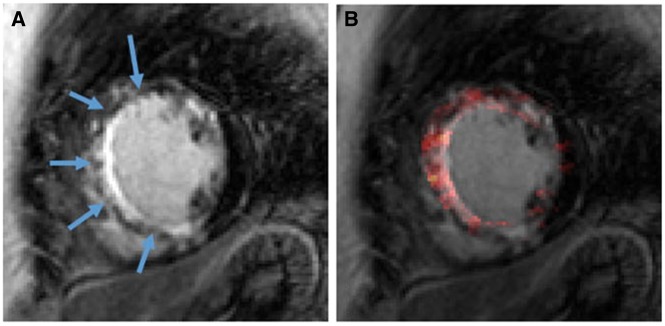
Subendocardial LGE demonstrating extensive infarct tissue (*A*) overlaid with ^18^F-Fluoride uptake indicating myocardial microcalcification (*B*). Reproduced with permission from ref. 60.

Another study demonstrated greater ^18^F-Fluoride uptake compared with non-culprit lesions on PET-MR following percutaneous revascularization.^60^ Interestingly, ^18^F-Fluoride uptake was demonstrated within infarcted myocardium, depicted from scar tissue, and in coronary plaque.^60^ (*Figure **2*).

#### Future outlook

Clinical studies of PET-MR thus far have demonstrated techniques to localize plaque rupture, although imaging was performed following acute cardiac events. Of much greater clinical relevance is the ability to localize and identify vulnerable plaques *prior* to rupture. Hybrid PET-MR imaging is well suited to meet this challenge: anatomical detail and plaque characterization can be obtained from MRI and combined with inflammatory atherosclerotic plaque activity from PET. Furthermore, emerging MRI motion models may be applied at multiple time points to motion-correct PET data and MRI data simultaneously without additional radiation.^20^ Emerging radiotracers (*Table **2*) such as ^18^F-Fluoride and ^68^Ga-DOTATATE may be evaluated with developing MRI motion correction techniques for precise anatomical identification of high-risk coronary plaques *prior* to rupture. This may allow targeted selection of high-risk patients for aggressive risk factor modification or revascularization and prevent adverse outcome.

## Cardiac sarcoidosis

**Figure 3:**
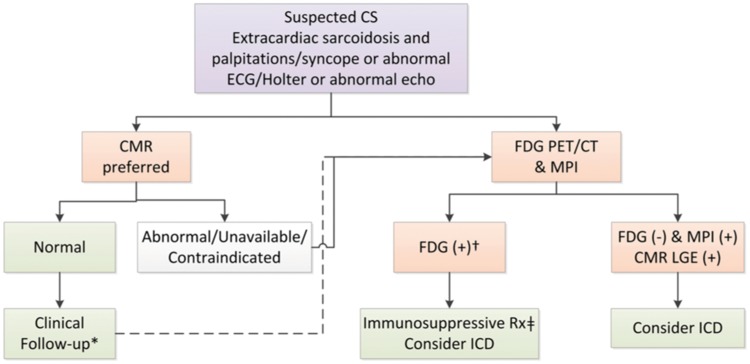
Recommended strategy for non-invasive imaging for assessment of patients with suspected cardiac sarcoidosis. Reproduced with permission from ref. 62.

Sarcoidosis is a multisystem granulomatous disorder that causes arrhythmia, conduction disease, cardiac failure, or sudden cardiac death. No universally accepted diagnostic test exists for cardiac sarcoidosis and sensitivity of endomyocardial biopsy is around 20%.^61^ Diagnosis of cardiac sarcoidosis is challenging, and multimodality imaging is recommended with MRI and PET in a recent international position paper (*Figure **3*).^62^

### MRI

Most commonly reported MRI findings in cardiac sarcoidosis include LGE of the mid-wall/epicardium of basal septum and lateral wall, and subendocardial and transmural enhancement.^63^ LGE in patients with cardiac sarcoidosis was an independent predictor for ventricular tachycardia and death.^64^^,^^65^ Patients (*n* = 61) with cardiac sarcoidosis (biopsy confirmed or clinical criteria) were found to have greater T1, T2, and ECV values compared with volunteers.^66^ A reduction in native T1 and T2 values was reported in 18 patients following anti-inflammatory therapy.^67^ Parametric mapping may serve as an imaging biomarker of disease activity and inflammation and guide anti-inflammatory therapy rather than LGE, which indicates fibrosis and may represent late sequelae or non-active disease.

### PET

^18^F-FDG lends itself well to identification of inflammatory lesions in active sarcoidosis as there is increased glucose utilization from macrophage activity. Careful patient preparation with low-carbohydrate diet and fasting prior to imaging is essential to avoid false positive results.^68^ PET imaging for sarcoidosis includes imaging with a perfusion radiotracer and ^18^F-FDG uptake with three patterns recognized: normal perfusion and no ^18^F-FDG uptake, abnormal perfusion or ^18^F-FDG uptake, and abnormal perfusion and metabolism.^69^ A perfusion-metabolism mismatch (reduced perfusion and increased ^18^F-FDG uptake) was associated with increased risk of cardiac death or ventricular arrhythmia.^69^

A meta-analysis demonstrated sensitivity of 89% and specificity of 78% for ^18^F-FDG to detect cardiac sarcoidosis compared with Japanese Ministry of Health and Welfare (JMHW) criteria.^70^

### PET-MR

Several studies have utilized PET-MR in patients with suspected cardiac sarcoidosis.

In a study of 51 patients, the sensitivities for cardiac sarcoidosis detection of PET and MRI alone were 85% and 82%, respectively, which improved to 94% with hybrid PET-MR using the JMHW criteria as reference standard in patients with suspected cardiac sarcoidosis.^71^ There was poor agreement between regions of high ^18^F-FDG uptake and LGE, although this is not an unexpected finding as the two modalities measure different entities of disease activity, and this study highlights the complementary information acquired from both modalities. At 2-year follow up, cardiac right ventricular PET involvement and presence of LGE were independent predictors of adverse events.^71^

**Figure 4:**
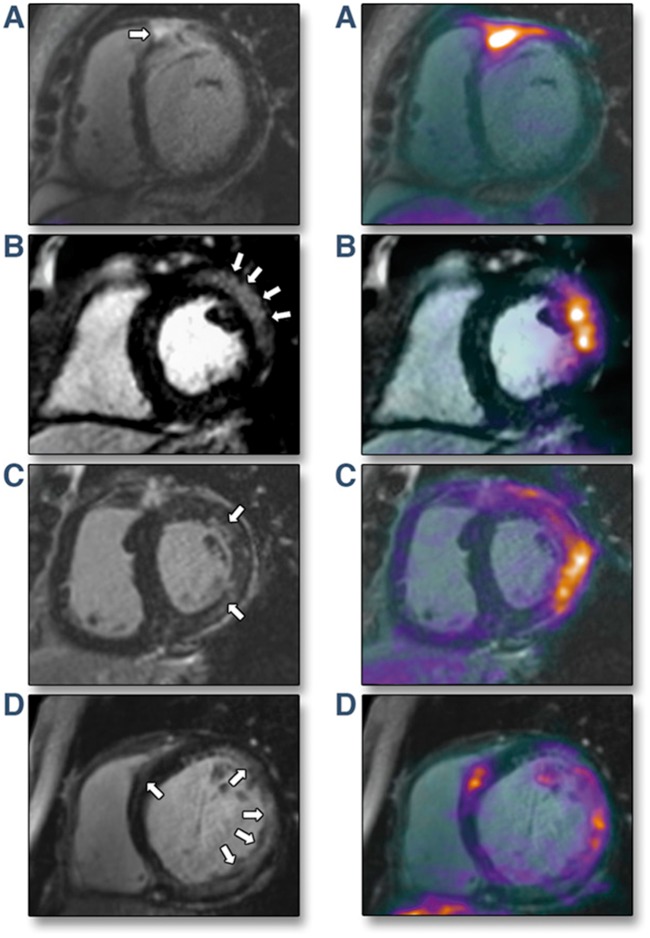
Imaging active cardiac sarcoidosis. *Left panel:* Late gadolinium enhancement (LGE) MRI. *Right panel*: Hybrid LGE and ^18^F-fluorodeoxyglucose (^18^F-FDG). (*A*) Subepicardial LGE in basal anteroseptum extending into right ventricular free wall and increased ^18^F-FDG uptake at same region on fused PET-MR. (*B*) Subepicardial LGE in the basal anterolateral wall with increased ^18^F-FDG uptake co-localizing to identical region on PET-MR. (*C*) Patchy midwall LGE in anterolateral wall with matched increased ^18^F-FDG uptake on PET-MR. (*D*) Multifocal LGE in lateral wall with matched increased ^18^F-FDG uptake on PET-MR. Reproduced with permission from ref. 72.

In another study, PET-MR was used in 25 patients with suspected active sarcoidosis, defined as positive LGE and ^18^F-FDG uptake (*Figure **4*).^72^ Higher ^18^F-FDG SUV outperformed T2-mapping by MRI (area under the curve 0.98 vs. 0.75, respectively) for detection of active sarcoidosis.^72^ This study demonstrated the utility of hybrid imaging to differentiate active cardiac sarcoidosis from non-active disease with an alternative classification, that may be more clinically meaningful compared with current established diagnostic criteria.

#### Future outlook

Hybrid PET-MR combines two powerful imaging modalities to obtain complementary information on distinct pathological processes in cardiac sarcoidosis: ^18^F-FDG PET delineates acute myocardial inflammation from macrophage activity, whilst MRI reveals regional motion wall abnormalities from granuloma formation, fibrosis from LGE, oedema from T2, and changes in myocardial structure with parametric mapping. The combination of precisely coregistered data in a single scan may be used to devise and validate much needed accurate diagnostic criteria for cardiac sarcoidosis. Furthermore, hybrid PET-MR may provide unique insights into the pathophysiology of cardiac sarcoidosis and serve as an imaging biomarker to guide anti-inflammatory therapy. Hybrid PET-MR may also derive perfusion-metabolism information from MRI perfusion data and ^18^F-FDG activity at reduced radiation burden.

## Myocarditis

Myocarditis is an inflammatory condition that causes chest pain, acute or chronic heart failure, life threatening arrhythmias, or cardiogenic shock.^73^ MRI is now commonly used in the diagnosis of myocarditis and to differentiate from myocardial infarction, while PET can also detect acute inflammation in myocarditis.

### MRI

MRI provides important information in patients with myocarditis: increased signal intensity with T2-weighted imaging in acute myocarditis results from oedema, early gadolinium enhancement (EGE) demonstrates capillary leakage and LGE identifies infiltration and scar, typically in the inferolateral walls and less frequently in the anteroseptum.^74^

The ‘Lake Louise’ Criteria (which are currently being revised) requires two of three positive MRI features—EGE, LGE, and T2 imaging.^74^ In addition, native T1-mapping may be used and in a study 50 patients had superior diagnostic performance compared with T2-weighted imaging in patients with suspected myocarditis.^75^ Furthermore, native T1 provided incremental diagnostic value and outperformed the original Lake-Louise criteria in acute myocarditis.^76^

### PET

^18^F-FDG is highly sensitive to metabolically active inflammation.^77^^18^F-FDG had sensitivity and specificity of 100% for detection of endomyocardial biopsy proven active myocarditis in patients when performed within 14 days of onset.^78^ Another study demonstrated increased uptake of ^68^Ga-DOTA-TOC in patients when PET-CT performed within 3–10 days of symptom onset.^79^

### PET-MR

**Figure 5:**
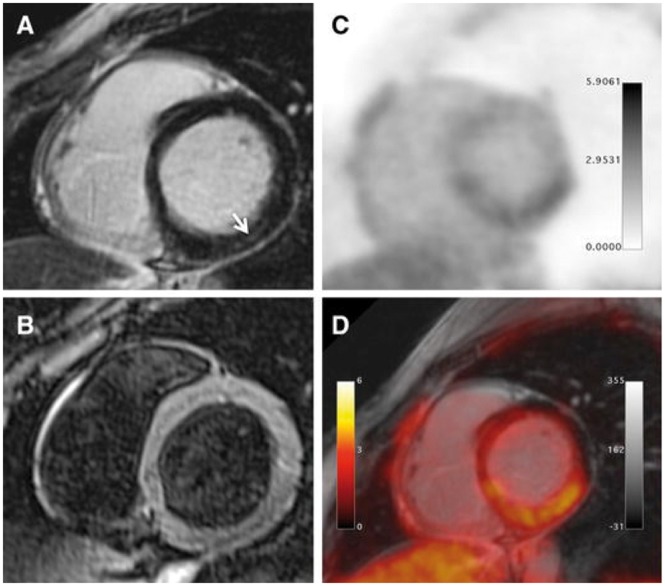
PET-MR images in myocarditis. Inferolateral wall mid-wall fibrosis (MRI) (*A*), homogenous T2 signal intensity (MRI) (*B*). Increased ^18^F-FDG signal in the inferolateral wall (PET) (*C*). Fused PET-MR with increased ^18^F-FDG uptake at the site of inferolateral LGE (*D*). Reproduced with permission from ref. 80.

In the only study of simultaneous PET-MR in patients (*n* = 55) with suspected myocarditis, there was good agreement between ^18^F-FDG and T2 and/or LGE (*Figure *5).^80^ However, no EGE imaging was performed, nor did patients undergo endomyocardial biopsy as a reference standard. ^18^F-FDG may provide additive information in patients with diffuse myocardial damage not detectable by MRI in diffuse inflammation or before myocyte necrosis.

#### Future outlook

A study of combined parametric mapping and ^18^F-FDG has yet to be performed, which will be of greater value for cross-validation. Moreover, PET-MR may aid in staging and risk stratification of patients with myocarditis by combining inflammatory activity with PET ^18^F-FDG or novel tracers such as ^68^Ga-DOTA-TOC with MRI assessment of fibrosis and oedema. In addition, PET-MR may guide biopsy of inflammatory foci with high metabolic activity through precise anatomical localization by MRI.^81^

## Amyloidosis

Cardiac amyloidosis is an infiltrative condition characterized by deposition of beta-pleated sheets and causes restrictive cardiomyopathy. Differentiation of light-chain amyloid (AL) or wild type/familial-related amyloid (ATTR) is important as treatment and prognosis differ.

### MRI

Typical MRI findings in amyloidosis include symmetric or asymmetric left ventricular thickening, biatrial enlargement, and pleural effusions. LGE shows global transmural or subendocardial LGE in non-coronary artery territory distribution^82^ and had sensitivity of 85% and specificity of 92% for detection of cardiac amyloid in a recent meta-analysis.^83^ Transmural LGE was an independent marker for mortality in patients with cardiac amyloid.^84^ Native T1-values are elevated in ATTR amyloid patients compared with volunteers and patients with hypertrophic cardiomyopathy.^85^

### PET

^11^C-Pittsburgh B, a radiotracer for detection of beta-amyloid deposits in Alzheimer’s patients, was detected in 13/15 patients with biopsy confirmed cardiac amyloid, whereas none was detected in patients without amyloid.^86^^18^F-Florbetapir also detects beta-amyloid plaques and a pilot study demonstrated greater uptake in patients with cardiac amyloid compared with controls.^87^^,^^88^

### PET-MR

Only one published study to date has explored PET-MR in cardiac amyloid. Seven patients with AL or ATTR amyloid and 7 controls underwent PET-MR imaging.^89^ T1 values were similar between AL and ATTR amyloid. Fused and co-registered images allowed precise measurement of tissue ^18^F-Fluoride activity in areas of myocardial amyloid deposition depicted by LGE. ATTR amyloid patients had higher tissue-to-background ratios of ^18^F-Fluoride uptake compared with AL amyloid patients or controls.^89^

#### Future outlook

Hybrid PET-MR may have a role in the future to discriminate AL and ATTR amyloid heart disease, although larger clinical studies are required prior to consideration as a routine clinical application.

## Anderson-Fabry disease

Anderson-Fabry disease (AFD), an X-linked lysosomal storage disorder characterized by deficiency of alpha-galactosidase, results in glycosphingolipids accumulation and may cause left ventricular hypertrophy (LVH), myocardial fibrosis, systolic and diastolic dysfunction, arrhythmia, conduction disease, and sudden death.^90^ Early diagnosis is important as enzyme replacement therapy (EZT) can reverse disease progression.^91^

### MRI

Typical MRI findings in AFD include concentric, asymmetric, or apical LVH and mid-wall fibrosis of the basal inferolateral wall.^92^^,^^93^ Native T1-mapping can add to the detection of AFD since lipids lower T1-values and accurately discriminate AFD from other causes of LVH such as hypertrophic cardiomyopathy, aortic stenosis, and hypertension.^94^

### PET

Two studies reported that AFD patients had lower MPR with ^15^O-Water compared with controls.^95^^,^^96^ This may relate to increased vascular resistance secondary to cardiac myocyte hypertrophy and fibrosis due to intracellular glycosphingolipids accumulation. Accumulating glycolipids may trigger an inflammatory response with pro-inflammatory cytokines and increased macrophage activity that eventually lead to fibrotic changes.^97^ PET may have a role to potentially detect inflammation at early stages of disease.

### PET-MR

In a study of 13 patients with AFD who underwent PET-MR, 5 patients had LVH and focal LGE fibrosis of which 3 had positive STIR and focal ^18^F-FDG uptake.^98^ The latter group had raised cardiac troponin levels, whilst those patients with negative STIR images did not have focal ^18^F-FDG uptake. The authors suggested PET-MR differentiated mature fibrosis or scar from fibrosis associated to active inflammation. However, this was a small sample size and several patients had homogenous ^18^F-FDG uptake, possibly indicating insufficient myocardial suppression.

#### Future outlook

PET-MR may aid diagnosis of AFD, provide insights into immunopathology of different disease stages and have potential as an imaging biomarker to guide EZT.

## Cardiac masses

In clinical practice, cardiac masses are primarily imaged with echocardiography and CT, while MRI and PET are reserved for further characterization.

### MRI

MRI can interrogate cardiac masses with T1-weighted black-blood imaging for mass localization, cine imaging for morphology, mobility and functional consequence, and tagging for assessment of attachments to other structures.^99^ T1 and T2-weighted imaging with/without fat suppression aids further tissue characterization.^99^ Furthermore, EGE allows thrombus detection, LGE allows assessment of extracellular matrix, whilst perfusion allows vascularity assessment. MRI has diagnostic accuracy of 92% for prediction of lesion type compared with histology findings.^100^ LGE can differentiate benign and malignant tumours with diagnostic accuracy of 79%.^101^

### PET

PET allows whole body evaluation of masses with ^18^F-FDG uptake for staging, monitoring therapy, and prognosis. Malignant tumours have greater ^18^F-FDG uptake compared with benign tumours.^102^ The diagnostic accuracy for detection of malignant lesions was 96%, with SUV_max_ cut-off of 3.5–4.0.^103^

### PET-MR

In a study of 20 patients who underwent PET-MR for cardiac mass assessment, ^18^F-FDG SUV_max_ was higher in malignant lesions and SUV_max_ cut-off of 5.2 provided 100% sensitivity and 92% specificity for malignancy detection.^104^ Similarly, a 100% sensitivity and 92% specificity was achieved for MRI to detect malignancy, using combined cine imaging, T1-weighted, T2-weighted, post-contrast T1-weighted images and presence of pericardial effusion.^104^ A hybrid approach of MRI and PET with SUV_max_ cut-off of 5.2 increased sensitivity and specificity to 100%. These are interesting findings, although first-pass perfusion, EGE or LGE imaging was not reported as part of the ‘MRI only’ classification, which may have otherwise increased diagnostic accuracy for MRI alone.

#### Future outlook

Hybrid PET-MR can offer additional information though precise co-registration and localization of masses to be undertaken, with detailed interrogation with high resolution T1 and T2-weighted images, parametric mapping, perfusion, LGE imaging and ^18^F-FDG uptake in one sitting that may improve diagnosis, staging and provide further clarity on disease activity.

## Future perspectives

PET-MR is an emerging modality gaining widespread interest for application to cardiovascular disease.

The ability to obtain multiple radiation-free MRI data to motion-correct PET data is highly attractive, and future research will focus on improved efficiency and reduction in acquisition time of MRI sequences to simultaneously enhance PET images, quantitative PET data as well as MRI data, particularly for coronary imaging and the application of emerging radiotracers.

AC has long been a challenge for PET-MR and novel methods to develop highly accurate AC maps such as with the inclusion of bone segmentation may be applied in future studies.^14^ Manual methods for misalignment of AC maps allows correction and studies thus far have demonstrated PET-MR provides comparable quantitative data to PET-CT. Another pressing challenge is a solution for AC map signal drop-out from metallic artefacts, which may become increasingly important in the future with emerging MRI compatible implantable cardiac devices at 3 T,^105^ the field strength of available clinical PET-MR systems.

PET-MR is an expensive modality that requires proximity to a cyclotron, specialist staff trained in PET and MRI for patient preparation, image acquisition, AC, post-processing, analysis and reporting. Simultaneous PET-MR imaging may improve patient workflow, with improved patient experience of a single scan. However, clear clinical indications for a hybrid PET-MR scan require evidence of incremental benefit compared with sequential imaging with separate MRI and PET scans. There is vast potential for application to research for cardiac PET-MR, particularly for coronary imaging which may pave the way for multicentre studies for identification of high-risk vulnerable plaques prior to rupture. With the current evidence available, clinical PET-MR may be well suited to conditions such as cardiac masses and suspected cardiac sarcoidosis where hybrid imaging offers precise co-registration and fusion of complementary information that may provide incremental value.

Once clear clinical indications and pathways are developed, these will need to be recognized as cost-effective for healthcare systems with a defined route for reimbursement.

## Conclusion

Hybrid PET-MR combines two powerful imaging modalities to fuse and co-register anatomical detail, tissue characterization, and metabolic data. Technical challenges with AC remain and there is large potential for motion correction of PET data from MRI data. Several studies thus far have focussed on cross-validation of techniques and emerging studies indicate incremental benefit beyond sequential MRI and PET imaging. Future large studies will determine incremental utility of combined rather than sequential imaging and whether PET-MR will be restricted to the research domain or cement itself in the clinical arena.
